# Association Between Perceived COVID‐19 Risk and Current Use of Any Nicotine Product Amongst Adolescents in Saudi Arabia

**DOI:** 10.1111/dar.70166

**Published:** 2026-05-10

**Authors:** Najim Z. Alshahrani, Abdullah M. Alarifi, Ibrahim Alasqah, Ahmed K. Shukri, Abdulrahman M. Albeshry, Mohannad A. Alzain, Abdullah Algarni, Mowffaq Mohammed Kalantan, Mohamed Terra, António Raposo, Nada Alqarawi, Sarah Almutairi, Mohamed Baklola

**Affiliations:** ^1^ Department of Family and Community Medicine, Faculty of Medicine University of Jeddah Jeddah Saudi Arabia; ^2^ Deputyship of Population Health, Ministry of Health Riyadh Saudi Arabia; ^3^ College of Medicine Alfaisal University Riyadh Saudi Arabia; ^4^ Department of Community, Psychiatric, and Mental Health Nursing, College of Nursing Qassim University Buraydah Saudi Arabia; ^5^ Nursing Services Department Qassim University Medical City Buraydah Saudi Arabia; ^6^ Department of Family Medicine, Faculty of Medicine King Abdulaziz University Jeddah Saudi Arabia; ^7^ Department of Family Medicine King Abdulaziz University Hospital, King Abdulaziz University Jeddah Saudi Arabia; ^8^ Family Medicine and Chronic Diseases Research Unit, King Fahd Medical Research Center King Abdulaziz University Jeddah Saudi Arabia; ^9^ Department of Medicine, College of Medicine University of Jeddah Jeddah Saudi Arabia; ^10^ Department of Family and Community Medicine, Faculty of Medicine University of Tabuk Tabuk Saudi Arabia; ^11^ Faculty of Medicine Mansoura University Mansoura Egypt; ^12^ CBIOS (Research Center for Biosciences and Health Technologies), ECTS (School of Health Sciences and Technologies) Lusófona University Lisboa Portugal

**Keywords:** adolescents, COVID‐19, nicotine, risk perception, tobacco use

## Abstract

**Introduction:**

The COVID‐19 pandemic created a unique context for tobacco‐related risk communication amongst adolescents. This study examined the link between COVID‐19–related smoking risk perceptions and current nicotine use amongst Saudi school adolescents.

**Methods:**

Data were drawn from the 2022 Saudi Arabia Global Youth Tobacco Survey, a nationally representative school‐based survey (overall response rate: 92.3%). The analytic sample included 4984 adolescents aged 13–15 years. The primary outcome was current use (≥ 1 day in the past 30 days) of any nicotine product, including cigarettes, shisha, smokeless tobacco, heated tobacco products and electronic cigarettes. Key exposures were beliefs about whether smoking affects COVID‐19 infection risk and illness severity. Survey‐weighted logistic regression estimated crude and adjusted odds ratios (aOR), adjusting for sex, grade, weekly spending, parental and peer smoking and school tobacco education. Product‐specific sensitivity analyses were performed.

**Results:**

Overall, 10.7% of adolescents reported current nicotine use. Adolescents who believed smoking reduced the risk of COVID‐19 infection had higher odds of nicotine use compared with those who believed it increased risk (aOR = 3.77; 95% CI 2.52–5.65). Similarly, perceiving that smoking reduced COVID‐19 severity was associated with higher odds of use (aOR = 2.34; 95% CI 1.49–3.67). In sensitivity analyses, associations for perceived reduced infection risk were observed for both cigarette smoking (aOR = 4.44; 95% CI 2.83–6.99) and non‐cigarette nicotine use (aOR = 4.09; 95% CI 2.96–5.66).

**Discussion and Conclusions:**

Beliefs that smoking lowers COVID‐19 risks are strongly linked to adolescent nicotine use, underscoring the need for accurate risk communication in youth tobacco prevention.

## 1 Introduction

Adolescent nicotine use remains a pressing global public health concern, with sustained uptake of cigarettes, shisha, e‐cigarettes and other tobacco products despite decades of prevention efforts [1, 2, 3, 4]. Young people are particularly susceptible to tobacco initiation due to factors such as peer influence, misperceptions of harm and targeted marketing strategies [5, 6]. In Saudi Arabia, recent surveys indicate that more than one in 10 adolescents report current (past 30‐day) use of nicotine products, including cigarettes, waterpipe (shisha) and electronic cigarettes [7], underscoring the urgent need to better understand the drivers of initiation and continued use in this vulnerable age group [1].

The COVID‐19 pandemic created a unique context for tobacco risk communication. Emerging epidemiological evidence suggested that smoking was associated with increased risk of severe COVID‐19 outcomes, including hospitalisation, intensive care unit admission and mortality [8, 9]. Biological mechanisms, such as impaired pulmonary immune response, increased airway inflammation and impaired mucociliary clearance, provide biological plausibility for worse respiratory outcomes amongst people who smoke [8, 9]. However, conflicting early reports and widespread misinformation during the early stages of the pandemic contributed to public uncertainty regarding whether smoking increased susceptibility to infection or the severity of illness [8]. While public health messaging emphasised respiratory harms, little is known about how adolescents interpreted this information and whether their beliefs about smoking and COVID‐19 risk were associated with nicotine use behaviours.

Risk perception is a well‐established determinant of health behaviour. Evidence from international studies amongst adults suggests that perceptions of smoking‐related COVID‐19 risk influence tobacco‐related behaviours, including smoking reduction and quit attempts [10]. Adults who believe smoking increases the risk of COVID‐19 infection or severity are more likely to attempt quitting, while those who downplay or deny these risks are more likely to persist in tobacco use [10, 11]. However, adolescent populations may respond differently due to developmental, cognitive and social factors [12, 13]. To date, limited research has examined how adolescents conceptualise the relationship between smoking and COVID‐19, particularly in the Middle East and North Africa region, where youth tobacco use remains a growing public health concern, and the uptake of alternative nicotine products such as waterpipe and electronic cigarettes has increased in recent years.

Understanding how health crises influence adolescents' health‐related beliefs and behaviours is critical not only for tobacco control but also for broader pandemic preparedness and health communication strategies [14, 15]. Addressing this gap, the present study analyses nationally representative data from the 2022 Saudi Arabia Global Youth Tobacco Survey (GYTS). Specifically, we investigate whether adolescents' perceptions of COVID‐19 infection risk and illness severity associated with smoking are linked to their current use of nicotine products, including cigarettes, shisha, smokeless tobacco, heated tobacco products and e‐cigarettes. By examining these associations in a nationally representative sample, this study provides insights into the relationship between pandemic‐related smoking risk perceptions and adolescent nicotine use, with implications for youth‐focused tobacco prevention strategies.

## 2 Methods

### 2.1 Study Design and Population

This study utilised data from the 2022 Saudi Arabia GYTS, a nationally representative, school‐based cross‐sectional survey designed to monitor tobacco use and related behaviours amongst adolescents [16]. The GYTS follows a globally standardised methodology developed by the World Health Organization (WHO) and the US Centres for Disease Control and Prevention [14]. In 2022, Saudi Arabia conducted its fourth GYTS, targeting school‐going adolescents in Grades 1–3 of intermediate school, corresponding approximately to ages 13–15 years [14]. This study is reported in accordance with the STROCSS 2025 checklist [17].

### 2.2 Sampling and Participants

A two‐stage stratified cluster sampling design was applied. In the first stage, schools were selected with probability proportional to enrollment size. In the second stage, classes within selected schools were randomly chosen, and all students in these classes were eligible to participate. Data were collected using an anonymous, self‐administered questionnaire distributed during regular school sessions. A total of 6983 students completed the survey. For this analysis, we restricted the sample to adolescents aged 13–15 years who had complete data on nicotine use and COVID‐19–related beliefs. After applying these criteria, 4984 respondents were included in the final analytic sample. The overall response rate reached 92.3%.

### 2.3 Measures

#### 2.3.1 Outcome Variable

The primary outcome was current use of any nicotine product, defined as self‐reported use of cigarettes, shisha, smokeless tobacco, heated tobacco products or e‐cigarettes on at least 1 day in the past 30 days. A binary variable was constructed, classifying respondents as adolescents who reported current nicotine use (1), defined as reporting use of any nicotine product in the past 30 days, and adolescents who did not report nicotine use (0).

For sensitivity analyses, two additional product‐specific outcomes were constructed. Current cigarette smoking was defined as smoking cigarettes on at least 1 day in the past 30 days. Current non‐cigarette nicotine use was defined as use of shisha, other smoked tobacco products, smokeless tobacco, heated tobacco products or e‐cigarettes on at least 1 day in the past 30 days. These product‐specific outcomes were examined to assess whether associations between COVID‐19‐related smoking perceptions and nicotine use were specific to combustible cigarette smoking or extended to other nicotine products. Based on prior evidence, the following covariates were included as potential confounders: sex, school grade, weekly spending money, parental smoking status, peer smoking and whether the respondent reported receiving school‐based education on the dangers of tobacco [3, 5, 11, 12, 14].

### 2.4 Statistical Analysis

Descriptive statistics were computed to summarise sample characteristics by current nicotine use. Differences between groups were tested using design‐based Pearson chi‐squared tests. Survey‐weighted logistic regression was used to estimate crude and adjusted odds ratios (OR) for the associations between each belief variable (perceived risk and perceived severity) and current nicotine use. All analyses accounted for the complex sampling design using the svyset command in Stata 18. The primary sampling unit, stratification variable and final sampling weights were specified according to the official GYTS guidance [16].

Four survey‐weighted logistic regression models were estimated. Model 1 was an unadjusted (crude) model. Model 2 was a multivariable main‐effects model adjusted for sex, grade level, weekly spending money, parental smoking, peer smoking and exposure to tobacco education. Model 3 extended Model 2 by including interaction terms between perceived COVID‐19 infection risk and peer smoking. Model 4 consisted of sex‐stratified versions of the main‐effects model. As a sensitivity analysis, we re‐estimated the adjusted main‐effects model (Model 2) separately for current cigarette smoking and current non‐cigarette nicotine use. This approach allowed us to evaluate whether associations between COVID‐19‐related smoking perceptions and nicotine use were product‐specific or reflected broader nicotine‐related behaviours. The same survey‐weighted modelling framework and covariate adjustments were applied. The extent of missing data for all primary variables is presented in Table S2. Analyses were conducted using complete‐case observations for the variables included in each model. Statistical significance was defined as a two‐sided *p* < 0.05. Statistical significance was defined as a two‐sided *p* < 0.05.

### 2.5 Ethical Considerations

This study used anonymised, publicly available data from the WHO's Noncommunicable Disease Microdata Repository. As the analysis was conducted using secondary data without identifiable personal information, additional ethical review was not required.

## 3 Results

### 3.1 Prevalence of Nicotine Use

Amongst the 4984 adolescents aged 13 to 15 years included in the analysis, 534 participants (10.7%) reported current use of at least one nicotine product within the past 30 days.

### 3.2 Sociodemographic and Environmental Correlates

Table 1 summarises participant characteristics by nicotine use status. Current nicotine use was significantly associated with male sex, higher grade level and greater weekly spending. Adolescents with smoking parents and those reporting peer smoking were substantially more likely to report current nicotine use, with peer smoking demonstrating the strongest gradient. Adolescents who reported current nicotine use more frequently reported having been taught about the dangers of tobacco compared with adolescents who did not report nicotine use. Perceptions of COVID‐19 risk related to smoking also differed significantly between groups. A smaller proportion of adolescents who reported current nicotine use believed that smoking increased the risk or severity of COVID‐19, whereas risk‐minimising beliefs (‘less at risk’ or ‘no difference’) were more common amongst adolescents who reported nicotine use (*p* < 0.001 for both infection risk and severity).

**TABLE 1:** Adolescent characteristics by current use of any nicotine product.

Characteristics	No current nicotine use (*n* = 4450)	Current nicotine use (*n* = 534)	All (*n* = 4984)	*p*
Sex, *n* (%)				**< 0.001**
Male	2255 (50.7)	316 (59.2)	2571 (51.6)	
Female	2195 (49.3)	218 (40.8)	2413 (48.4)	
Grade, *n* (%)				**< 0.001**
1st Intermediate	1348 (30.3)	126 (23.6)	1474 (29.6)	
2nd Intermediate	1779 (40.0)	204 (38.2)	1983 (39.8)	
3rd Intermediate	1323 (29.7)	204 (38.2)	1527 (30.6)	
Weekly spending, *n* (%)				**< 0.001**
I usually don't have	992 (22.3)	119 (22.3)	1111 (22.3)	
Less than 30 SAR	1591 (35.8)	148 (27.7)	1739 (34.9)	
30–49 SAR	914 (20.5)	90 (16.9)	1004 (20.1)	
50–99 SAR	565 (12.7)	95 (17.8)	660 (13.2)	
100 SAR or more	388 (8.7)	82 (15.4)	470 (9.4)	
Parental smoking, *n* (%)				**< 0.001**
Neither	3669 (82.5)	354 (66.3)	4023 (80.7)	
Both	116 (2.6)	48 (9.0)	164 (3.3)	
Father	500 (11.2)	93 (17.4)	593 (11.9)	
Mother	24 (0.5)	15 (2.8)	39 (0.8)	
I don't know	141 (3.2)	24 (4.5)	165 (3.3)	
Close friends smoke, *n* (%)				**< 0.001**
None of them	3905 (87.8)	299 (56.0)	4204 (84.4)	
Some of them	426 (9.6)	152 (28.5)	578 (11.6)	
Most of them	101 (2.3)	39 (7.3)	140 (2.8)	
All of them	18 (0.4)	44 (8.2)	62 (1.2)	
Taught dangers of tobacco, *n* (%)				**< 0.001**
Yes	1576 (35.4)	241 (45.1)	1817 (36.5)	
No	1820 (40.9)	181 (33.9)	2001 (40.2)	
Do not know	1054 (23.7)	112 (21.0)	1166 (23.3)	
Perceived risk of COVID‐19 from smoking				**< 0.001**
More at risk	3452 (77.6)	345 (64.6)	3797 (76.1)	
Less at risk	166 (3.7)	76 (14.2)	242 (4.9)	
No difference	832 (18.7)	113 (21.2)	945 (19.0)	
Perceived severity of COVID‐19 from smoking				**< 0.001**
More severe	3560 (80.0)	376 (70.4)	3936 (79.0)	
Less severe	194 (4.4)	55 (10.3)	249 (5.0)	
No difference	696 (15.6)	103 (19.3)	799 (16.0)	

*Note:* Bold values indicate statistical significance at *p* < 0.05.

### 3.3 Beliefs About Smoking and COVID‐19 Infection Risk

To quantify the associations observed descriptively in Table 1, survey‐weighted logistic regression models were estimated, examining perceived COVID‐19 infection risk from smoking and current nicotine use.

In crude analyses (Model 1), adolescents who believed that smoking reduced their risk of contracting COVID‐19 had higher odds of nicotine use compared with those who believed it increased risk (OR = 4.65, 95% confidence interval [CI] 3.25–6.65, *p* < 0.001). Those who believed smoking made no difference also had elevated odds (OR = 1.36, 95% CI 1.03–1.80, *p* = 0.031).

After adjustment for sex, grade, weekly spending, parental smoking, peer smoking and exposure to tobacco education (Model 2), the association for the ‘less at risk’ group remained statistically significant (aOR = 3.77, 95% CI 2.52–5.65, *p* < 0.001). The association for the ‘no difference’ group was attenuated and not statistically significant (aOR = 1.20, 95% CI 0.92–1.57, *p* = 0.172).

### 3.4 Beliefs About Smoking and COVID‐19 Severity

**FIGURE 1:**
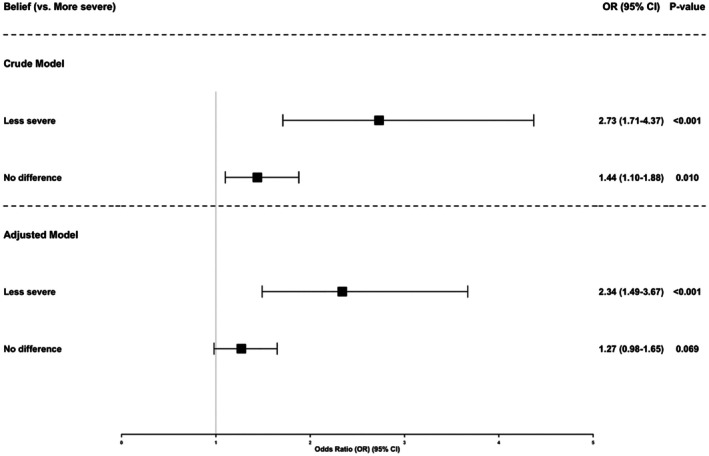
Crude (Model 1) and adjusted main‐effects (Model 2) odds ratios for current nicotine use by perceived COVID‐19 severity. ‘More severe’ was the reference category. The adjusted model includes sex, grade, weekly spending, parental and peer smoking and tobacco education exposure. Model 2 adjusted for sex, grade, weekly spending, parental smoking, peer smoking and tobacco education exposure. No interaction terms included.

Figure 1 presents associations between perceived COVID‐19 severity from smoking and current nicotine use. In crude models (Model 1), adolescents who believed smoking made COVID‐19 less severe had higher odds of nicotine use compared with those who believed it increased severity (OR = 2.73, 95% CI 1.71–4.37, *p* < 0.001). Adolescents who perceived no difference in severity also had elevated odds (OR = 1.44, 95% CI 1.10–1.88, *p* = 0.010).

After adjustment for sociodemographic and environmental covariates (Model 2), the association for the ‘less severe’ group remained statistically significant (aOR = 2.34, 95% CI 1.49–3.67, *p* < 0.001), whereas the association for the ‘no difference’ group was attenuated and did not reach statistical significance (aOR = 1.27, 95% CI 0.98–1.65, *p* = 0.069).

### 3.5 Multivariable Survey‐Weighted Logistic Regression

**FIGURE 2:**
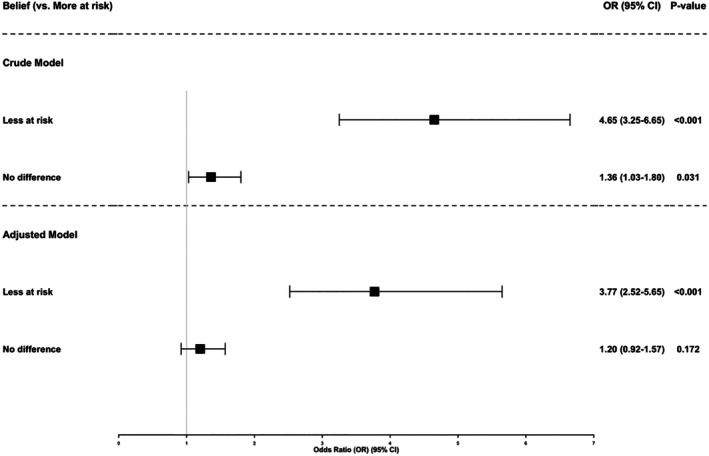
Crude (Model 1) and adjusted main‐effects (Model 2) odds ratios for current nicotine use by perceived COVID‐19 infection risk. ‘More at risk’ was the reference category. The adjusted model includes sex, grade, weekly spending money, parental smoking, peer smoking and school‐based tobacco education exposure. Model 2 adjusted for sex, grade, weekly spending, parental smoking, peer smoking and tobacco education exposure. No interaction terms included.

Table 2 presents results from the extended multivariable model (Model 3), which includes interaction terms between perceived COVID‐19 infection risk and peer smoking. Because interaction terms modify the interpretation of main effects, the resulting odds ratios represent conditional associations and therefore differ numerically from the adjusted main‐effects model presented in Figure 2.

**TABLE 2:** Survey‐weighted logistic regression model including interaction between perceived COVID‐19 infection risk and peer smoking (Model 3).

Predictor	OR	95% CI	*p*
Perceives ‘no difference’ versus ‘more risk’	0.91	0.65–1.27	0.2
Perceives ‘less risk’ versus ‘more risk’	3.69	2.27–6.00	< 0.001^a^
Peer smoking (any)	3.37	2.45–4.62	< 0.001^a^
Parental smoking	1.49	1.09–2.04	0.01^a^
Taught about tobacco dangers	1.40	1.16–1.69	0.001^a^
Interaction:No‐difference × peer smoking	2.17	1.19–3.96	0.01^a^
Interaction: Less‐risk × peer smoking	1.18	0.60–2.32	0.1

*Note:* Results are from a survey‐weighted logistic regression model adjusted for sex, grade, weekly spending money, parental smoking, peer smoking and school‐based tobacco education. ‘More risk’ is the reference category for perceived COVID‐19 infection risk. Estimates are conditional on the reference category of peer smoking due to the inclusion of interaction terms.

Abbreviations: CI, confidence interval; OR, odds ratio.

^a^ Significant values when *p*‐value < 0.05.

In this model, perceiving that smoking made adolescents less at risk of COVID‐19 infection remained associated with higher odds of nicotine use (aOR = 3.69, 95% CI 2.27–6.00, *p* < 0.001). Peer smoking was strongly associated with nicotine use, as was parental smoking. A statistically significant interaction was observed between perceiving ‘no difference’ in infection risk and peer smoking (aOR = 2.17, 95% CI 1.19–3.96, *p* = 0.01). All covariates included in the model are presented in Table 2.

### 3.6 Sex‐Stratified Models

Sex‐stratified survey‐weighted logistic regression models were estimated to examine whether associations differed by sex (Table 3). All covariates included in the main‐effects model were retained in both sex‐specific models.

**TABLE 3:** Sex‐stratified adjusted main‐effects models (Model 4) predicting current nicotine use.

Predictor	OR	95% CI
*A. Males*		
Less risk	3.19	2.10–4.83
Peer smoking	3.02	2.10–4.35
High spending (≥ 100 SAR)	2.25	1.51–3.34
*B. Females*		
Less risk	5.18	2.55–10.51
Peer smoking	6.16	4.35–8.72
Taught about tobacco dangers	1.70	1.39–2.07

*Note:* Estimates are from survey‐weighted logistic regression models stratified by sex and adjusted for grade level, weekly spending and parental smoking. Models are stratified by sex and adjusted for grade level, weekly spending, parental smoking, peer smoking and tobacco education exposure. No interaction terms included.

Abbreviations: CI, confidence interval; OR, odds ratio.

Amongst males, perceiving that smoking reduced COVID‐19 infection risk was associated with higher odds of nicotine use (aOR = 3.19, 95% CI 2.10–4.83). Peer smoking and higher weekly spending were also associated with nicotine use.

Amongst females, perceiving reduced infection risk was associated with higher odds of nicotine use (aOR = 5.18, 95% CI 2.55–10.51). Peer smoking was strongly associated with nicotine use, and exposure to tobacco education was also associated with use. All model estimates, including non‐significant associations, are presented in Table 3.

### 3.7 Sensitivity Analyses by Product Type

To assess whether associations were specific to cigarette smoking or extended to other nicotine products, we re‐estimated the adjusted main‐effects model separately for current cigarette smoking and current non‐cigarette nicotine use. Perceiving that smoking reduced the risk of COVID‐19 infection remained strongly associated with both cigarette smoking (aOR = 4.44, 95% CI 2.83–6.99) and non‐cigarette nicotine use (aOR = 4.09, 95% CI 2.96–5.66). In contrast, perceiving no difference in infection risk was significantly associated with cigarette smoking (aOR = 1.80, 95% CI 1.21–2.67) but not with non‐cigarette nicotine use (aOR = 1.22, 95% CI 0.96–1.53). Full sensitivity analysis results are provided in supplementary Tables S1 and S2.

These findings suggest that strong COVID‐19‐related smoking risk misperceptions are associated with broader nicotine use behaviours, while weaker risk minimisation may be more specific to combustible cigarette smoking.

## 4 Discussion

This study examined the association between adolescents' COVID‐19‐related smoking risk perceptions and current nicotine use using nationally representative data from Saudi Arabia. Adolescents who believed that smoking reduced the risk of COVID‐19 infection or severity had higher odds of current nicotine use compared with those who believed smoking increased risk. These associations persisted after adjustment for sociodemographic factors, parental and peer smoking, weekly spending and tobacco education exposure. Interaction analyses indicated that the association between perceived infection risk and nicotine use differed according to peer smoking, and sex‐stratified models showed stronger associations amongst girls than boys.

These associations remained robust even after adjusting for sociodemographic variables, spending power, exposure to parental and peer smoking, and school‐based tobacco education. Notably, the strength of the association was greatest amongst adolescents who reported that smoking made COVID‐19 infection less likely, highlighting a potentially concerning pattern of risk minimisation amongst adolescents who reported nicotine use. This aligns with concerns raised early in the pandemic that youth may be particularly susceptible to misinformation, and that evolving scientific guidance about COVID‐19 may not have reached all audiences with equal clarity or relevance [18, 19, 20].

The significance of these findings lies not only in their statistical strength but also in their broader implications for understanding the behavioural legacy of the COVID‐19 pandemic. During public health emergencies, messaging around risk is often directed at adult populations, with less attention given to how adolescents interpret and internalise such information [21]. Our results suggest that for some adolescents, the pandemic may have introduced or reinforced distorted beliefs about tobacco's impact on health—beliefs that, in turn, may have served to justify or rationalise nicotine use. In contexts such as Saudi Arabia, where youth smoking remains a persistent public health challenge and where diverse tobacco products, including shisha and e‐cigarettes, are widely accessible [1, 2], the emergence of pandemic‐related misperceptions may have further complicated efforts to prevent initiation and promote cessation amongst young people.

Our study contributes to a growing literature grounded in health psychology and behavioural science, which identifies risk perception as a key driver of individual behaviour [22, 23, 24]. According to frameworks such as the Health Belief Model and the Theory of Planned Behaviour, adolescents are more likely to engage in health‐compromising behaviours when they perceive the risks as low or irrelevant [25, 26]. In this context, beliefs that smoking does not increase vulnerability to COVID‐19, or even reduces it, may lower the psychological barriers to experimentation and regular use. Adolescents may interpret pandemic‐related health messages differently from adults due to developmental, cognitive and social factors. Peer norms and social influences play a particularly strong role in shaping adolescent health behaviours, including tobacco use, and may influence how risk information is interpreted within social networks [27, 28].

While adult studies suggest that heightened perception of COVID‐19 risk may motivate cessation attempts, adolescents may process such messages through the lens of peer norms, perceived invulnerability and social identity formation [13]. In the present study, adolescents who reported that smoking reduced COVID‐19 infection risk or severity had substantially higher odds of nicotine use, even after adjustment for parental and peer smoking. These findings illustrate how pandemic‐related health messages may not uniformly translate into protective behaviours amongst youth. Instead, adolescents who perceive the risks as minimal or who reinterpret risk information in ways that align with existing behaviours, may be less responsive to conventional public health messaging. Our findings support this interpretation: even after adjusting for the strongest known predictors of adolescent nicotine use, including peer smoking and parental behaviour, beliefs about COVID‐19 severity remained independently associated with current use. These findings indicate that COVID‐19‐related smoking risk perceptions were associated with nicotine use even after adjustment for measured social and environmental factors such as peer and parental smoking.

Adolescents' perceptions of smoking and COVID‐19 risk may also have been shaped by informal information environments during the pandemic. During COVID‐19, young people increasingly relied on social media platforms and peer networks to access and share health information, which created conditions for rapid dissemination of both accurate information and misinformation [18]. Studies have shown that health‐related misinformation circulating online can influence risk perceptions and health behaviours, particularly amongst younger populations who frequently encounter health information through social media channels [18, 20]. In the context of smoking and COVID‐19, widely circulated claims suggesting that nicotine might protect against infection or reduce disease severity may have contributed to confusion about the actual health risks. Such informal information flows, combined with peer discussions and evolving scientific messaging during the early stages of the pandemic, may therefore have reinforced risk‐minimising beliefs amongst some adolescents [18].

Beyond the crude associations, the multivariable analyses show evidence that misperceptions about COVID‐19 risks have an independent effect on adolescent nicotine use. Even after accounting for traditional determinants such as peer and parental smoking, grade level and spending money, adolescents who believed smoking reduced their risk of COVID‐19 infection remained over three times more likely to use nicotine products [29]. In the current study, a statistically significant interaction was observed between peer smoking and perceived COVID‐19 infection risk, indicating that the association between risk perception and nicotine use differed according to peer smoking status [30]. These findings underscore that risk perceptions during a health crisis do not merely coexist with nicotine use but may actively interact with social contexts to shape behaviour.

The sex‐stratified findings also add important nuance, indicating that pandemic‐related misperceptions may not affect all adolescents equally. In the sex‐stratified analyses conducted in this study, the association between believing smoking reduced COVID‐19 risk and nicotine use was stronger amongst girls than boys, with more than a fivefold increase in the odds of current use [31]. Peer smoking also had a disproportionately large impact on girls, emerging as the strongest predictor in the study. These patterns may reflect gendered differences in susceptibility to social cues, health beliefs or risk communication, and they highlight the importance of tailoring prevention strategies [29, 31]. Interventions that challenge misinformation and strengthen critical appraisal of health information may therefore require gender‐sensitive approaches to address the distinct pathways through which boys and girls engage with nicotine products. These findings are consistent with other recent studies amongst adolescents in Saudi Arabia, which have shown that nicotine use behaviours are strongly shaped by social influences, peer dynamics and exposure to tobacco control environments, highlighting the importance of both social and policy‐level determinants in adolescent tobacco prevention [32, 33].

The findings of this study have important implications for tobacco control and risk communication amongst adolescents. They show that the effects of health crises such as COVID‐19 extend beyond immediate outcomes and can shape long‐term beliefs and behaviours. Public health messages aimed at discouraging smoking during pandemics should be tailored for adolescents, clearly explaining the link between tobacco use and respiratory vulnerability in a developmentally appropriate and culturally relevant way. The persistence of misperceptions after the pandemic also suggests that corrective communication may be needed during the recovery period. In addition, the results support integrating tobacco education into broader health literacy curricula that help young people critically assess health information in uncertain contexts.

### 4.1 Strengths and Limitations

This study has several strengths. It uses nationally representative data from the Global Youth Tobacco Survey, collected with a rigorous two‐stage sampling design and standardised instruments. The analysis is grounded in behavioural theory and includes adjustment for key confounders such as sex, grade, spending power, parental and peer smoking and exposure to tobacco education. The outcome measure also captures a wide range of nicotine products, reflecting the diversity of adolescent nicotine use in the region.

This study also has several limitations. First, its cross‐sectional design precludes establishing causality between COVID‐19–related beliefs and nicotine use. Second, data were based on self‐reported behaviours and perceptions, which may be subject to recall or social desirability bias. Third, the analysis was restricted to school‐attending adolescents, potentially limiting generalisability to out‐of‐school youth. Fourth, the survey did not capture the influence of media exposure or misinformation, which may have shaped risk perceptions during the pandemic. Despite these limitations, the use of nationally representative data provides valuable insights into adolescent beliefs and nicotine use in Saudi Arabia.

## 5 Conclusions

This study demonstrates that adolescents in Saudi Arabia who believe smoking reduces the risk or severity of COVID‐19 are significantly more likely to use nicotine products. Misperceptions about the relationship between smoking and respiratory infections appear to influence behaviour, even after accounting for demographic and social factors. These findings highlight the critical role of health communication in shaping adolescent risk perception during public health crises. Targeted, age‐appropriate messaging that corrects misconceptions and strengthens risk literacy should be integrated into school‐based prevention programmes and national tobacco control strategies. Addressing these gaps will be essential not only for reducing adolescent nicotine use but also for enhancing resilience against misinformation in future pandemics.

## Author Contributions

**Najim Z. Alshahrani:** conceptualisation. **Najim Z. Alshahrani**, **Abdullah M. Alarifi**, **Ahmed K. Shukri** and **Abdulrahman M. Albeshry:** methodology. **Najim Z. Alshahrani** and **António Raposo:** validation. **Najim Z. Alshahrani** and **Abdullah M. Alarifi:** formal analysis. **Najim Z. Alshahrani**, **Abdullah M. Alarifi**, **Ibrahim Alasqah**, **Ahmed K. Shukri**, **Abdulrahman M. Albeshry**, **Mohannad A. Alzain**, **Abdullah Algarni**, **Mowffaq Mohammed Kalantan**, **Mohamed Terra**, **António Raposo**, **Nada Alqarawi**, **Sarah Almutairi** and **Mohamed Baklola:** investigation. **Najim Z. Alshahrani:** resources. **Najim Z. Alshahrani:** data curation. **Najim Z. Alshahrani**, **Abdullah M. Alarifi**, **Ahmed K. Shukri**, **Abdulrahman M. Albeshry:** writing – original draught preparation. **Najim Z. Alshahrani**, **Abdullah M. Alarifi**, **Ibrahim Alasqah**, **Ahmed K. Shukri**, **Abdulrahman M. Albeshry**, **Mohannad A. Alzain**, **Abdullah Algarni**, **Mowffaq Mohammed Kalantan**, **Mohamed Terra**, **António Raposo**, **Nada Alqarawi**, **Sarah Almutairi** and **Mohamed Baklola:** writing – review and editing. **Najim Z. Alshahrani** and **António Raposo:** visualisation. **Najim Z. Alshahrani** and **António Raposo:** supervision. **Najim Z. Alshahrani** and **António Raposo:** project administration. **Najim Z. Alshahrani**, **Ibrahim Alasqah**, **António Raposo**, **Nada Alqarawi** and **Sarah Almutairi:** funding acquisition. All authors have read and agreed to the published version of the manuscript.

## Funding

The authors have nothing to report.

## Ethics Statement

This study used anonymised, publicly available data from the WHO's Noncommunicable Disease Microdata Repository. As the analysis was conducted using secondary data without identifiable personal information, additional ethical review was not required.

## Consent

The authors have nothing to report.

## Conflicts of Interest

The authors declare no conflicts of interest.

## Supporting information


**Table S1:** Sensitivity results.
**Table S2:** Missing observations for primary outcome, exposure and covariate variables (*N* = 6983).

## Data Availability

The data that support the findings of this study are available from the corresponding author upon reasonable request.
