# Supplementary material for: Association Between Perceived COVID‐19 Risk and Current Use of Any Nicotine Product Amongst Adolescents in Saudi Arabia
Source: Drug Alcohol Rev. 2026 May 10;45:e70166. doi: 10.1111/dar.70166 (PMC13158642; doi:10.1111/dar.70166)
Supplement: Supplementary file 1 — Table S1: Sensitivity results. Table S2: Missing observations for primary outcome, exposure and covariate variables (N = 6983). [file DAR-45-0-s001.docx]

**Table S1. Sensitivity results**

1. Cigarette Smoking Outcome

| **Exposure** | **aOR** | **95% CI** |
| --- | --- | --- |
| Less risk | 4.44 | 2.83–6.99 |
| No difference | 1.80 | 1.21–2.67 |

1. Non-Cigarette Nicotine Outcome

| Exposure | aOR | 95% CI |
| --- | --- | --- |
| Less risk | 4.09 | 2.96–5.66 |
| No difference | 1.22 | 0.96–1.53 |

**Table S2.** Missing observations for primary outcome, exposure, and covariate variables (N = 6,983)

| Variable | Missing (n) | Missing (%) |
| --- | --- | --- |
| Any nicotine use | 803 | 11.5 |
| Cigarette smoking (past 30 days) | 315 | 4.5 |
| Non-cigarette nicotine use | 726 | 10.4 |
| Perceived COVID-19 infection risk (SAR92) | 121 | 1.7 |
| Perceived COVID-19 severity (SAR93) | 132 | 1.9 |
| Sex | 85 | 1.2 |
| Grade | 91 | 1.3 |
| Weekly spending money | 68 | 1.0 |
| Parental smoking | 28 | 0.4 |
| Peer smoking | 41 | 0.6 |
| Taught dangers of tobacco | 145 | 2.1 |

*Missing percentages are calculated using the full survey sample (N = 6,983). Analyses were conducted using complete-case observations for variables included in each model.*
